# Controlled Synthesis of Hollow α-Fe_2_O_3_ Microspheres Assembled With Ionic Liquid for Enhanced Visible-Light Photocatalytic Activity

**DOI:** 10.3389/fchem.2019.00058

**Published:** 2019-02-27

**Authors:** Hang Yin, YuLing Zhao, Qingsong Hua, Jianmin Zhang, Yuansai Zhang, Xijin Xu, Yunze Long, Jie Tang, Fengyun Wang

**Affiliations:** ^1^College of Physics and State Key Laboratory of Bio-Fibers and Eco-Textiles, Qingdao, China; ^2^National Engineering Research Center for Intelligent Electrical Vehicle Power System, Power & Energy Storage System Research Center, Qingdao University, Qingdao, China; ^3^School of Physics and Technology, University of Jinan, Jinan, China; ^4^1D Nanomaterials Group, National Institute for Materials Science, Tsukuba, Japan; ^5^Key Laboratory of Microelectronic Devices & Integrated Technology, Institute of Microelectronics, Chinese Academy of Sciences, Beijing, China

**Keywords:** α-Fe_2_O_3_, assembly, porous materials, ionic liquid, magnetic properties, visible light, photocatalytic activity

## Abstract

Porous self-assembled α-Fe_2_O_3_ hollow microspheres were fabricated via an ionic liquid-assisted solvothermal reaction and sequential calcinations. The concentration of the ionic liquid (1-butyl-3-methylimidazolium tetrafluoroborate [C_4_Mim]BF_4_) was found to play a crucial role in the control of these α-Fe_2_O_3_ hollow structures. Trace amounts ionic liquid was used as the soft template to synthesize α-Fe_2_O_3_ hollow spheres with a large specific surface (up to 220 m^2^/g). Based on time-dependent experiments, the proposed formation mechanisms were presented. Under UV light irradiation, the as-synthesized α-Fe_2_O_3_ hollow spheres exhibited excellent photocatalysis in Rhodamine B (RhB) photodegradation and the rate constant was 2–3 times higher than α-Fe_2_O_3_ particles. The magnetic properties of α-Fe_2_O_3_ hollow structures were found to be closely associated with the shape anisotropy.

## Introduction

Nanostructured oxides with a variety of useful functionalities are widely applied in photocatalysis, energy storage, etc. (Guo et al., 2017; Li et al., 2017; Zhao et al., 2017, 2018). In particular, hematite (α-Fe_2_O_3_), an environmentally-friendly magnetic photocatalyst (E_g_ = 2.2 eV), has been identified as an important material because of its potential for a wide range of practical applications (Brezesinski et al., 2011; Yang et al., 2014; Zhou et al., 2014; Ma et al., 2018). Recently, self-assembled hematite with highly specific hollow nano/micro-structures and unique properties has emerged as being of great interest to material scientists. To date, various self-assembled hollow α-Fe_2_O_3_ nano/micro-structures have been prepared by different synthetic techniques, including the two-step reaction process (Yu J. et al., 2009), hydrothermal (Xu et al., 2012), and solvothermal approaches (Song et al., 2012; Zhu et al., 2012), thermal oxidation at high temperature (Xie et al., 2010), etc. For example, Yu and co-workers successfully fabricated cage-like Fe_2_O_3_ hollow spheres and carbonaceous polysaccharide spheres were used as porous crystalline shell templates, followed by calcination at 500°C for 4 h. Song and co-workers proposed a hydrolysis route to synthesis Fe_2_O_3_ hierarchical hollow spheres, via a sodium dodecyl benzenesulfonate (SDBS)-assisted hydrolysis process. However, these template methods often suffer from a problem: it is difficult to remove the template and surface-active agent completely. Furthermore, template removal always leads to additional problems in sample morphology. Therefore, it is still necessary to develop a simple route to synthesizing assembled hollow α-Fe_2_O_3_ structures.

Ionic liquids (ILs), or “designer liquids,” are known for their superior properties such as recyclability and stable chemical properties. They are used extensively in catalysis, separations, electrochemistry, and especially nanochemistry (Welton, 1999; Wasserscheid and Keim, 2000; Seddon, 2003). The most important advantage of ILs is that they can form extended hydrogen bond systems in the liquid, thereby enabling highly-structured nanostructures (Mele et al., 2003). This special performance can be used as the “entropic driver” for synthesis of self-assembled nanostructures. It has been proved that ILs can be used not only as solvents, but also as templates for preparing nanomaterials with improved properties (Endres et al., 2003; Cooper et al., 2004; Liu et al., 2006). For instance, a variety of nano/micro-structures have been synthesized via ILs, especially assembled hollow structures such as Bi_4_O_5_Br_2_ and BiOBr spheres (Xia et al., 2011a; Mao et al., 2017) and α-Fe_2_O_3_ hollow polyhedral (Xu et al., 2013). These hollow structures may also exhibit unique properties for photocatalysis.

In this work, we report a simple and feasible ionic liquid-assisted solvothermal method to synthesize self-assembled α-Fe_2_O_3_ hollow microspheres. The ionic liquid [C_4_Mim]BF_4_ acts as a soft template can be easily removed by annealing in air. Compared with α-Fe_2_O_3_ particles, the as-synthesized α-Fe_2_O_3_ hollow spheres exhibit enhanced photocatalytic activity due to their porous self-assembly structure and higher surface area. As far as we known, α-Fe_2_O_3_ porous hollow microspheres with high surface area (above 200 m^2^/g) prepared via this ionic liquid-assisted solvothermal synthesis route is reported for the first time. Additionally, the possible formation mechanisms have also been proved. The as-prepared α-Fe_2_O_3_ samples exhibit ferromagnetic properties and can be recycled easily.

## Experimental

### Materials and Methods

#### Materials

Ferric chloride (FeCl_3_·6H_2_O, 99%) and ethylenediamine (EDA) were purchased from Tianjin Damao Chemical Co. The ionic liquid ([C_4_mim]BF_4_) was obtained from Beijing Solarbio Technology Co. Ethylene glycol (EG) was purchased from Tianjin Chemical Reagent Co. and distilled water was used throughout the experiment.

#### Synthesis

In a typical experimental process, FeCl_3_·6H_2_O (0.81 g) was dissolved into 60.0 mL of ethylene glycol (EG). Then, 0, 0.1, 0.2, 0.3, 0.4, and 0.5 mL of [C_4_Mim]BF_4_ were added to the above solution. After 2 h, 3 mL of ethylenediamine (EDA) was added into the mixture solution to form a well-distributed emulsion. After 1 h, the emulsion was transferred into a Teflon-sealed autoclave with a capacity of 100 mL, sealed and heated at 200°C for 20 h. After cooling to room temperature, the resultant precipitate was washed with deionized water and ethanol several times until the solution was neutral. After drying in a vacuum oven at 60°C for 6 h, the dried products were calcined in air at 250°C for 6 h. The reaction time was adjusted to study the mechanism.

### Materials Characterization

X-ray diffraction (XRD) patterns were obtained with a Rigaku RINT 2500 diffractometer using a Cu-Kα radiation source (λ = 1.5406 Å). The morphology and microstructure of the as-prepared samples were examined with scanning electron microscopy (SEM, JEOL 6500F). TEM and HRTEM images were recorded with a Tecnai accelerating voltage of 120 and 200 kV, respectively. The nitrogen adsorption/desorption isotherms at 77.35 K were collected on an AUTOSORB iQ-MP instrument after heating the samples at 150°C for 2 h. The surface areas were estimated using the Brunauer-Emmett-Teller (BET) method in the relative pressure range of 0.05–0.3 Pore size distributions were analyzed using nitrogen adsorption data in a Barrett-Joyner-Halenda (BJH) model. Magnetic properties of the as-synthesized samples were measured with a physical property measurement system (PPMS-9T, ever cool II, USA).

### Photocatalytic Reaction

20.0 mg of α-Fe_2_O_3_ photocatalyst catalyst and 50.0 mL of RhB dye aqueous solution (10.0 mg/L) were mixed in a Py”prex reactor and stirred in the dark for 30 min to reach a complete absorption/desorption equilibrium. Afterwards, the suspension was exposed to a 300 W Xenon lamp and stirred simultaneously. Subsequently, 3.0 mL of suspension was centrifuged at 5,000 rpm for 4 min to remove the photocatalyst. Finally, the concentration of RHB was monitored with a TU-1901 UV-vis spectrophotometer by monitoring the absorbance at 553 nm.

## Results and Discussion

### Structure and Morphology Characterization

**Figure 1:**
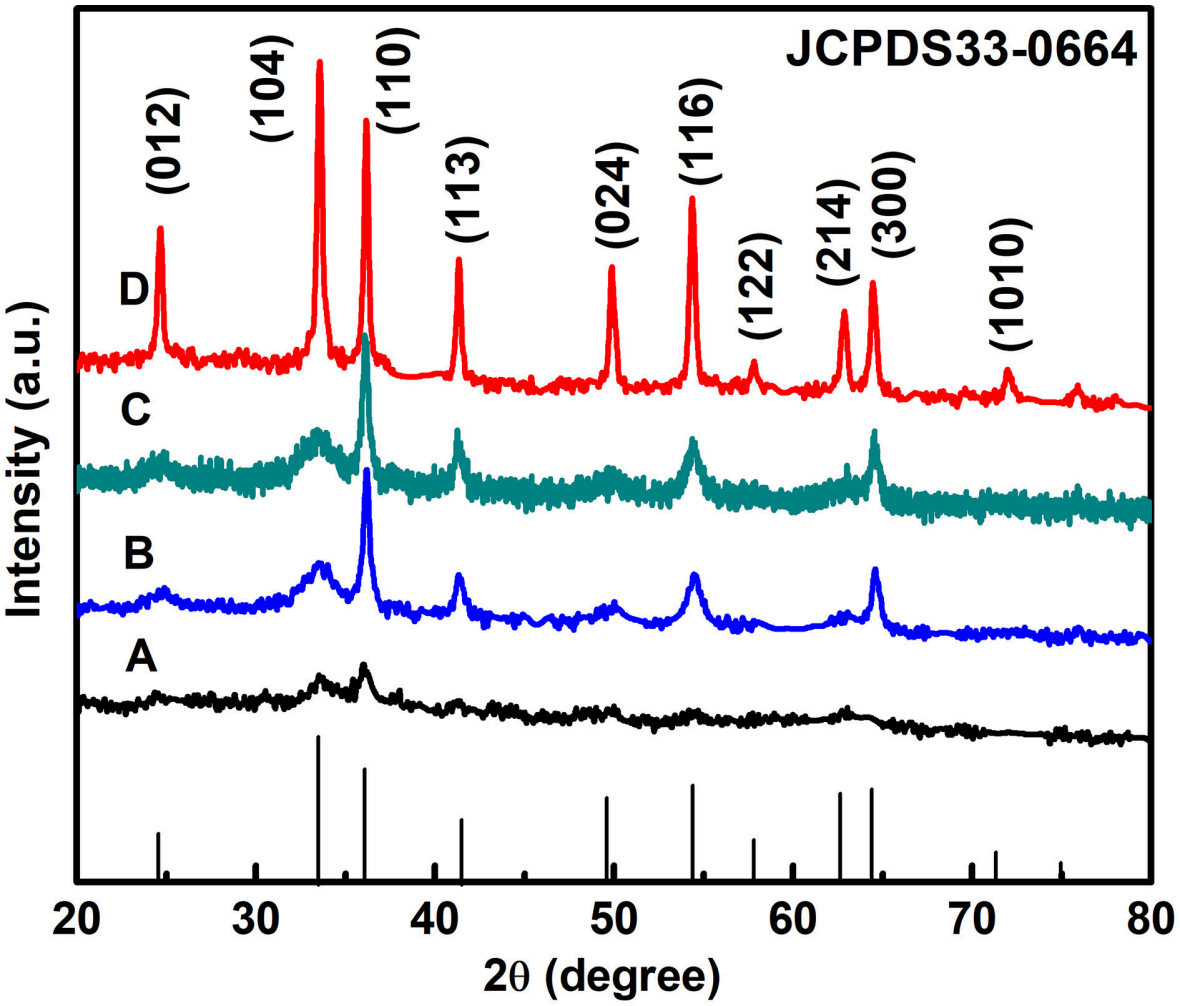
XRD patterns of the α-Fe_2_O_3_ with different amounts of [C_4_Mim]BF_4_: **(A)** 0 mL, **(B)** 0.1 mL, **(C)** 0.2 mL, and **(D)** 0.3 mL.

The purity and crystallinity of the as-prepared powder samples are measured by XRD (Figure 1). The XRD shows that all diffraction peaks can be indexed in α-Fe_2_O_3_ (JCPDS 33–0664) with structural parameters of a = b = 5.038 Å, c = 13.749 Å, α = β = 90°, and γ = 120°. No other peaks are observed, demonstrating that the α-Fe_2_O_3_ products have high-purity and a single phase. Therefore, we can infer that anions and cations of the [C_4_Mim]BF_4_ are not doped into the α-Fe_2_O_3_ lattice. As the [C_4_Mim]BF_4_ addition increases, the peak intensities of α-Fe_2_O_3_ also gradually increase, indicating that sample (D) has the highest degree of crystallization. This result proves that the addition of ionic liquid can enhance the crystallization of as-synthesized materials. A similar phenomenon is also observed in the previous report (Xu et al., 2013).

According to the full width at half-maximum (fwhm) of the diffraction peaks, the average crystallite size of the α-Fe_2_O_3_ nanoparticles and microspheres ([C_4_Mim]BF_4_ = 0.1, 0.2, 0.3 mL) can be estimated from the Scherrer equation to be about 40.2, 84.6, 101.2, and 198.5 nm, respectively.

$$\begin{array}{l} D_{hkl}=K\lambda /\left(\beta_{hkl}\cos\theta_{hkl}\right) \end{array}$$

where *D*_*hkl*_ is the particle size perpendicular to the normal line of (*hkl*) plane, *K* is a constant (it is 0.9), β_*hkl*_ is the full width at half-maximum of the (*hkl*) diffraction peak, θ_*hkl*_ is the Bragg angle of (*hkl*) peak, and λ is the wavelength of X-ray.

**Figure 2:**
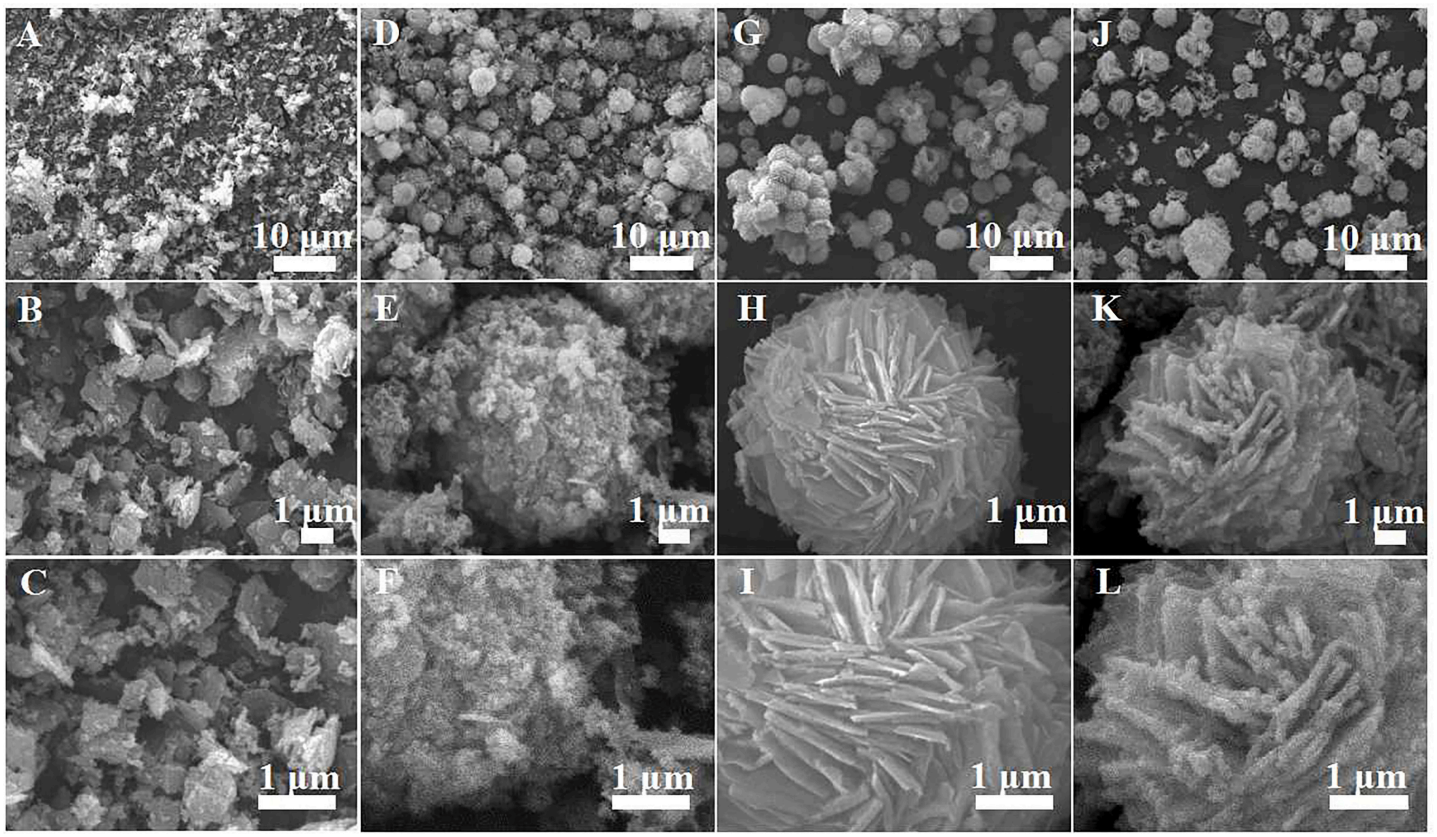
SEM images of α-Fe_2_O_3_ samples prepared with varying addition of [C_4_Mim]BF_4_ at 200°C for 20 h: **(A–C)** 0 mL, **(D–F)** 0.1 mL, **(G–I)** 0.2 mL, **(J–L)** 0.3 mL.

The morphology and structure of the as-prepared samples are studied by SEM and TEM Figure 2 shows the typical SEM micrographs of the α-Fe_2_O_3_ samples prepared with varying [C_4_Mim]BF_4_ additions at 200°C for 20 h. In the absence of [C_4_Mim]BF_4_ (Figures 2A–C), only α-Fe_2_O_3_ pseudo cubic particles are formed. The morphology and size of α-Fe_2_O_3_ particles are non-uniform and the alignment is disordered. As the amount of [C_4_Mim]BF_4_ increased from 0.1 to 0.3 mL, the as-prepared α-Fe_2_O_3_ changed from irregular particles to the uniform microspheres.

**Figure 3:**
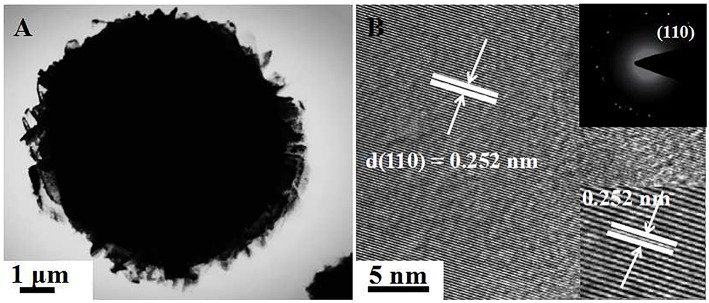
**(A)** TEM image of single sphere obtained at [C_4_Mim]BF_4_ = 0.3 mL, **(B)** HRTEM image. The inset shows the corresponding SAED pattern (up-right).

Figures 2D–F show the SEM image of [C_4_Mim]BF_4_ = 0.1 mL, which is composed of nanoparticles. When the addition of [C_4_Mim]BF_4_ is up to 0.2 mL, perfectly spherical α-Fe_2_O_3_ is formed. As shown in Figure 2G, some broken α-Fe_2_O_3_ microspheres reveal that the as-obtained α-Fe_2_O_3_ microspheres are of a hollow structure. Therefore, we can infer that [C_4_Mim]BF_4_ is used as a template in the process of α-Fe_2_O_3_ synthesis. The three-tiered organization of crystallites for these hollow dandelions is shown in the magnified image (Figure 2H). Numerous nanosheets are present on the surface of the dandelion spheres, with a puffy appearance and the average diameter being about 6.5 μm. From Figure 2I, we can see that individual nanosheets have an average size of about 100 nm, which is in agreement with the result calculated from Scherrer's formula. When the [C_4_Mim]BF_4_ amount increases to 0.3 mL (Figures 2J,H,L), the similar nanosheets stack up in three-dimensional ordered microspheres (5.0–7.0 μm) with a larger thickness (about 200 nm). Figure 3A shows the TEM image of an α-Fe_2_O_3_ microsphere. However, the structure in [C_4_Mim]BF_4_ = 0.3 mL demonstrates no internal void space. A high-resolution TEM (HRTEM) image (Figure 3B) exhibited lattice spacing of 0.252 nm, corresponding to the (110) lattice plane. Additionally, some examples of synthesizing nanostructured α-Fe_2_O_3_ using different kinds of ILs are given in Table 1.

**Table 1:** Comparison of different α-Fe_2_O_3_ samples synthase by this work and others reported in previous references.

**Reaction system**	**Ionic liquid**	**Morphology**	**Size**	**References**
Ethanol + NaOH	[OmMim]FeCl_4_	Microspheres	2–4 μm	Xu et al., 2013
water + NaOH	[BMim]Cl	Microcubes	600 nm	Lian et al., 2009
Water + CH-_3_COOK	[BMim]Cl	Mesoporous hollow Microspheres	1 μm	Lian et al., 2009
Urea + water	[C_4_Mim]BF_4_	Hollow polyhedral	0.5–1 μm	Xu et al., 2012
Urea +[C_12_Mim] Br	[C_12_Mim] Br	Nanorods	Diameter 30 nm Length 200 nm	Ping et al., 2017
EG + NaOH	[C_2_Mim] [C_2_OSO_3_]	Nanoparticles	12–47 nm	Shikha et al., 2015
EG + EDA	[C_4_Mim]BF_4_	Microspheres	5–7 μm	This work

Additionally, the self-assembly micron spheres are made up of a bunch of nanosheets, indicating that the spheres are porous in structure. It is striking that when the addition of [C_4_Mim]BF_4_ reaches to 0.4 mL, precipitation is very sparse. Until 0.5 mL, no precipitation synthesized after reaction. This phenomenon can be explained as follows: EG and ionic liquids are not mutually soluble, so the two-phase interface is formed. With an increase in the addition of ionic liquids, the disorder of atoms at the two-phase interface increases, resulting in a growing of interfacial energy, which lifts the nucleation resistance. Owing to large resistance, crystal cannot nucleate.

### Nitrogen Sorption

**Figure 4:**
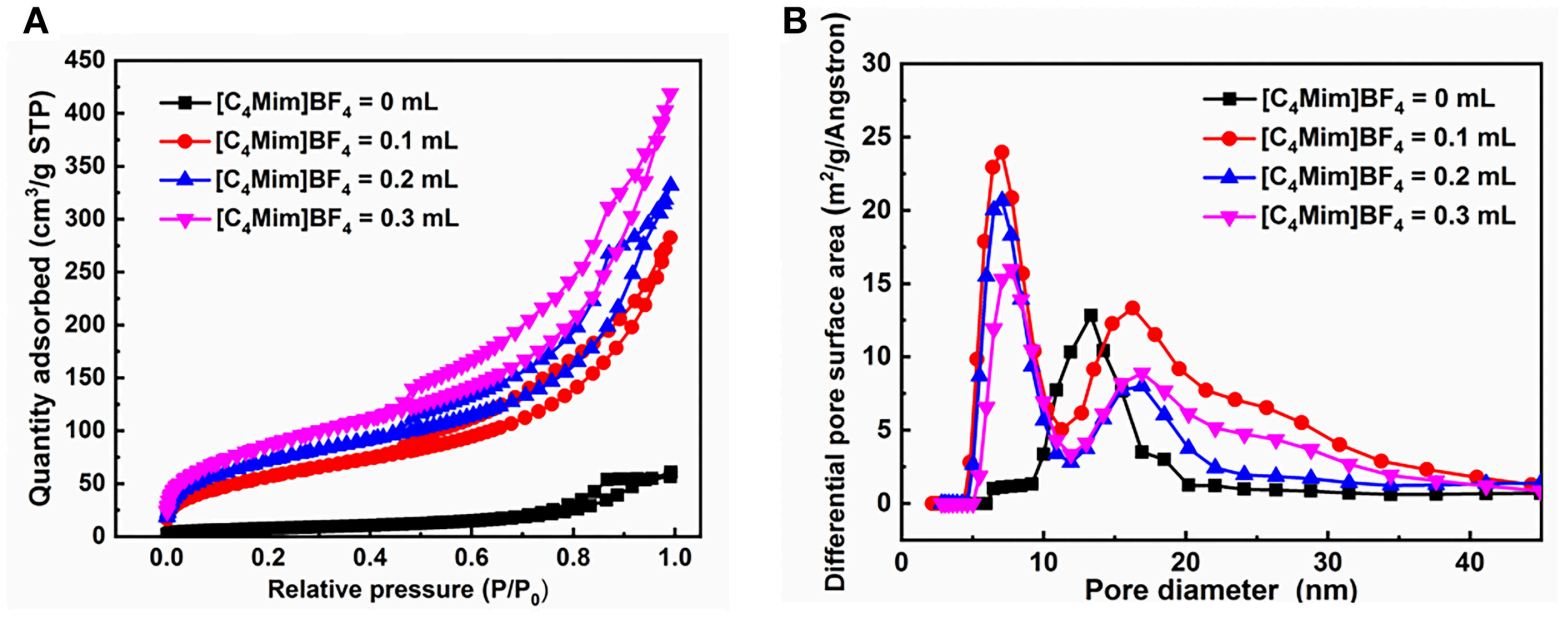
**(A)** Nitrogen adsorption-desorption isothermal, **(B)** the pore size distribution curve for the α-Fe_2_O_3_.

Figure 4 shows the nitrogen adsorption-desorption isotherms and pore size distributions of the as-prepared α-Fe_2_O_3_. The isotherms of α-Fe_2_O_3_ samples shown in Figure 4A are of type IV with a hysteresis loop from 0.5 to 1.0 (P/P_0_). The BET-specific surface areas of α-Fe_2_O_3_ hollow microspheres are calculated to be 183, 208, and 221 m^2^g^−1^, all larger than that of α-Fe_2_O_3_ synthesized without [C_4_Mim]BF_4_ (only 44 m^2^g^−1^). Correspondingly, the BJH pore size of the as-synthesized α-Fe_2_O_3_ ranges from 5.0 to 10.0 nm (Figure 4B). The smaller pore structures may arise from the crystal growth, and the larger pores ascribe to the stacking of the α-Fe_2_O_3_ nano-structures and the hollow structure. Additionally, the peaks of the pore sizes of the α-Fe_2_O_3_ move to the right after adding [C_4_Mim]BF_4_, showing that average pore size increases. From this result, we can infer that the crystallinity of the as-prepared α-Fe_2_O_3_ improved (Zhu et al., 2012), which is in agreement with the XRD analysis. Table 2 summarizes the mean pore size and S_BET_ of the as-obtained α-Fe_2_O_3_. It shows that with increasing additions of [C_4_Mim]BF_4_, S_BET_ increases dramatically. This result may be attributed to two factors: first, compared with irregular α-Fe_2_O_3_ nanoparticles or nanosheets, hollow assembly structures have more opportunities to participate in nitrogen adsorption. Without the [C_4_Mim]BF_4_, the specific surface area of α-Fe_2_O_3_ is only 44.0 m^2^g^−1^ (Xia et al., 2011b). Second, the presence of a large number of mesoporous structures (6–7 nm) leads to a large specific surface area.

**Table 2:** Brumaire–Emmett–Teller (BET) surface area and mean pore diameters of the as-prepared samples.

**Sample**	**Addition (mL)**	**BET surface areas (m^**2**^ g^**−1**^)**	**BJH pore size (nm)**
1	0	44	13.33
2	0.1	183	6.42
3	0.2	208	7.08
4	0.3	221	7.71

### Possible Formation Mechanism of α-Fe_2_O_3_ Hollow Spheres

**Figure 5:**
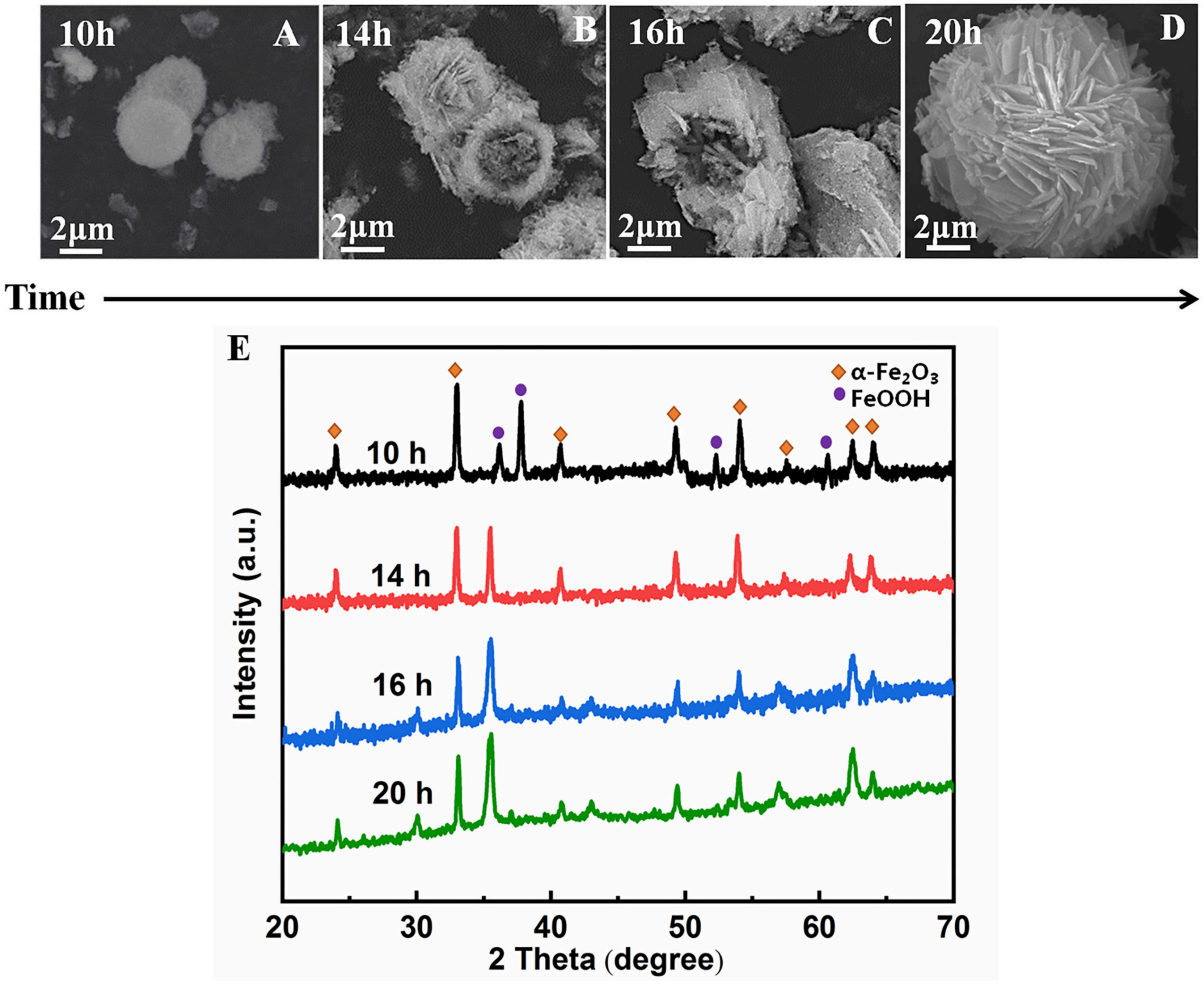
SEM images of α-Fe_2_O_3_ hollow microsphere structures synthesized in the presence of ionic liquid [C_4_Mim]BF_4_ = 0.2 mL at 200°C. **(A)**10 h, **(B)** 14 h, **(C)** 16 h, **(D)** 20 h. **(E)** XRD patterns of the α-Fe_2_O_3_ at different time.

To better understand the formation process of the α-Fe_2_O_3_ porous hollow spheres, time-dependent experiments were carried out. As shown in Figures 5A–D, representative SEM images at different time intervals are displayed. First, a hollow core-shell spherical structure was generated at 10 h. The mean diameter of the core is about 2.0 μm, which was further confirmed by the TEM image shown in Figure S1. Second, some microparticles were deposited on its surface at 14 h. Then, as the reaction time reached 16 h, some nanosheets began to cover the surface of the nuclei, indicating that deposition was still in progress. When the duration reached 20 h, a hollow sphere covered with a great number of nanosheets was obtained.

**Figure 6:**
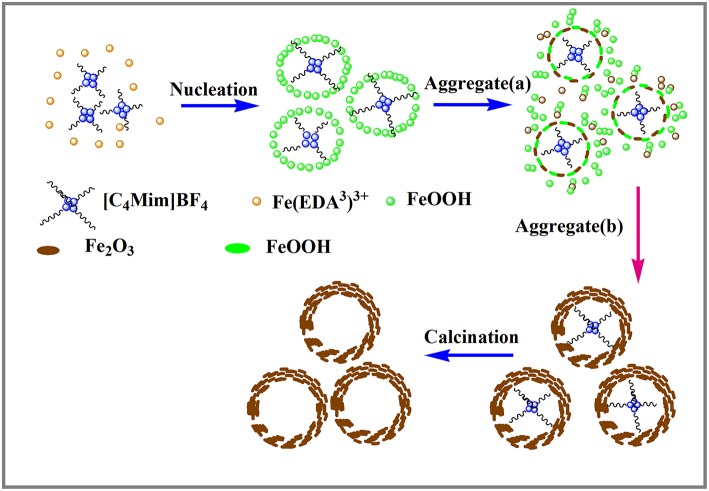
Schematic illustration of the formation of α-Fe_2_O_3_ hollow spheres.

Based on the above experimental results, the nucleation and growth mechanism for the microsphere in the two-phase system was proposed, as shown in Figure 6. First, Fe^3+^ reacted with EDA in EG-[C_4_Mim]BF_4_ solution to form a relatively stable complex ion of [Fe (EDA)^3^]^3+^ (Zhang et al., 2006). High temperature caused complex ions [Fe (EDA)^3^]^3+^ to decompose into FeOOH. Thus, FeOOH was first formed. Then, the FeOOH nanoparticles dissolved and reacted with the obtained Fe^2+^ in a [C_4_Mim]BF_4_ assisted system to form α-Fe_2_O_3_ nanoparticles. From Figure 5E, the peaks of FeOOH and α-Fe_2_O_3_ can be observed at 10 h. The formed α-Fe_2_O_3_ nanoparticles were unstable and had a tendency to form larger congeries, which may have been driven by the minimization of interfacial energy. That is why after 14 h, the sample only showed the crystal image of α-Fe_2_O_3_. However, EG solution has greater viscosity and fewer surface hydroxyls than the aqueous solution, resulting in kinetically slower nucleation and aggregation of nanocrystals, which lead to the formation of perfectly oriented assemblies by the adequate rotation to find the low-energy configuration interface (Zhu et al., 2013). A longer reaction time leads to directional alignment to generate spherical nuclei at 10 h. During the subsequent process, nanoparticles gradually assemble into nanosheets and stack on the surface of the template. The reason for the formation of nanosheets is that small particles gradually adhere to large particles to form thin sheets, which is commonly referred as Ostwald ripening. Finally, a specified morphology forms. Such a process is also observed in several other reports (Lou et al., 2006; Zhu et al., 2008; Yu X. et al., 2009). In this study, the adjacent primary ferric alkoxide nanocrystals have high activity due to their high surface energy. They further grow into nanosheets by directional aggregation, which greatly reduces the interface energy of small primary nanocrystals. Then, by directional attachment and self-assembly, the nanosheets gradually evolve into 3D flower-like superstructures. At the same time, it is combined with the mature process of Ostwald to form a favorable hollow structure.

In this formation process, the reaction time is one of the most important controlling factors. Apart from the reaction time, EDA is also a critical factor. EDA is used as a precipitant during this process. When a part of an EG molecule loses protons and coordinates with FeCl_3_ to form ferric alkoxide, H^+^ is produced in the reaction of EG with metal chloride. If H^+^ cannot be removed, the accumulation of H^+^ will inhibit the formation of further iron oxides. In this paper, EDA takes the lead to react with Fe^3+^ to form a stable complex and inhibit the formation of H^+^ in the reaction system.

### Enhancement of Photocatalytic Activity

**Figure 7:**
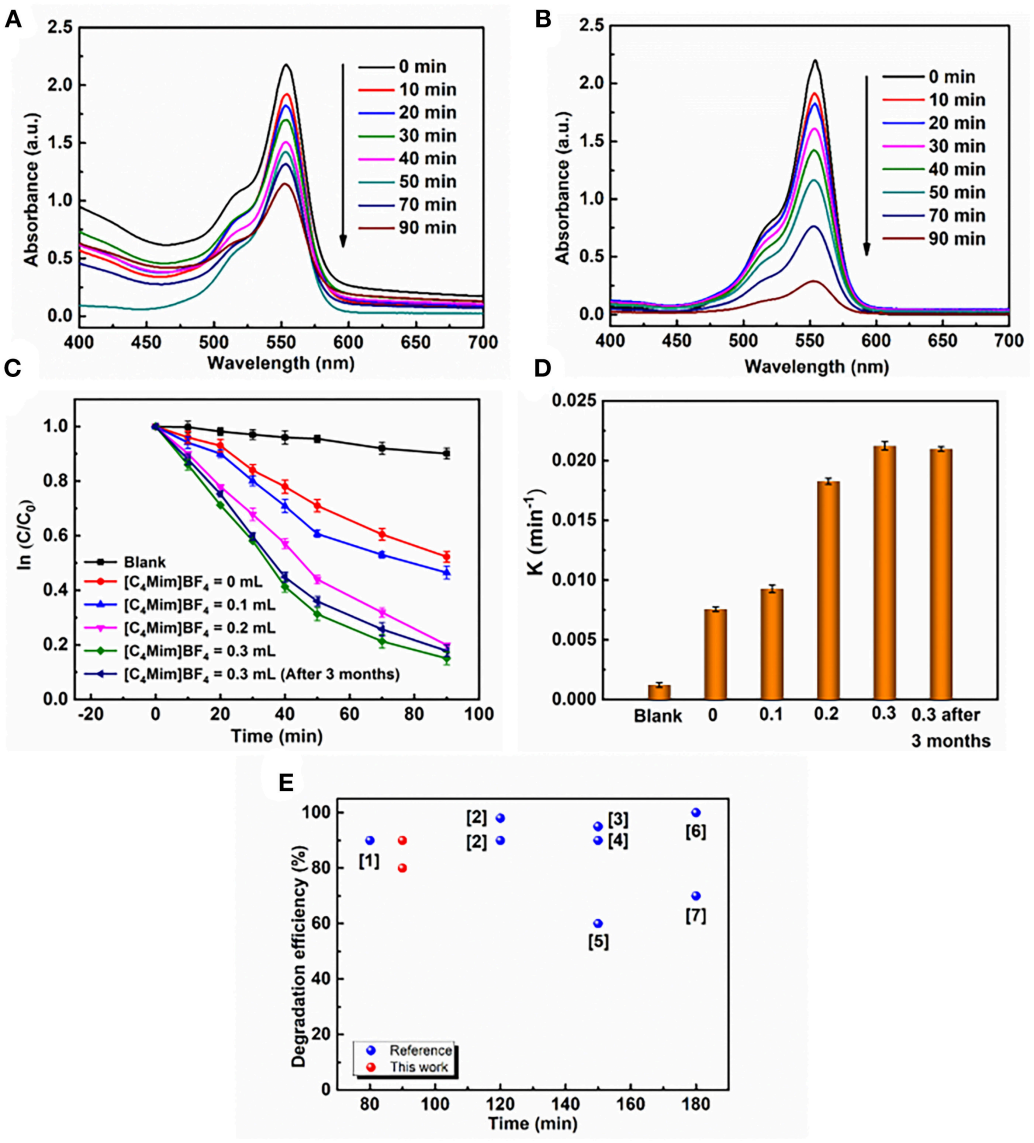
UV–vis absorption spectra during photocatalytic reaction of RhB over α-Fe_2_O_3_ with different [C_4_Mim]BF_4_ additions at different time intervals: **(A)** 0 mL, **(B)** 0.3 mL, **(C)** plots of ln(C/C_0_) against time t of various samples: without catalyst, the α-Fe_2_O_3_ hollow spheres prepared with addition = 0, 0.1, 0.2, and 0.3 mL, respectively. **(D)** Reaction kinetics of RhB degradation under visible light irradiation by α-Fe_2_O_3._ **(E)** Comparison of degradation efficiencies of different α-Fe_2_O_3_ samples during the RhB photodegradation driven by visible light. Red balls refer to the α-Fe_2_O_3_ samples prepared in this work and blue balls refer to the α-Fe_2_O_3_ samples reported in previous references. ([1] Xu et al., 2012; [2] (Pawar and Choi, 2015); [3] (Cai et al., 2014); [4] (Xu et al., 2012); [5] (Liang et al., 2014); [6] (Zhou et al., 2011); [7] (Wu et al., 2013)).

The photocatalytic degradation of RhB is monitored by measuring the absorption behavior of the solution at 553 nm. The evolutions of the spectrums are shown in Figure 7A for α-Fe_2_O_3_ synthesized without [C_4_Mim]BF_4_ and in Figure 7B for that synthesized with 0.3 mL [C_4_Mim]BF_4_. When the addition amount reaches 0.3 mL, 90% of RhB is decomposed after 90 min irradiation, while it is no more than 50% for the sample synthesized without [C_4_Mim]BF_4_. Thus, the use of [C_4_Mim]BF_4_ in the synthesis process effectively improves the photocatalytic performance. To clarify the effect of [C_4_mim]BF_4_, we figure out the rate constants of the degradation processes for the α-Fe_2_O_3_ synthesized with various concentrations of [C_4_Mim]BF_4_. To evaluate the reactivity, the apparent reaction rate constant (k) is calculated. Figure 7C shows ln (C/C_0_)—t plots for the α-Fe_2_O_3_ samples synthesized with different [C_4_Mim]BF_4_ amounts as well as the blank test. The rate constant *k* is 0.00756, 0.00927, 0.0183, 0.0212 min^−1^ for [C_4_Mim]BF_4_ = 0.0, 0.1, 0.2, and 0.3 mL, respectively, shown in Figure 7D. Thus, the photocatalytic performance is found to improve with increasing [C_4_Mim]BF_4_ addition amounts. As shown in Figure 7E, we carried out a coarse comparison of degradation efficiencies between this study and other studies based on the photodegradation of RhB by α-Fe_2_O_3_.

In addition to the high catalytic efficiency, the catalyst possessed robust stability. In our case, α-Fe_2_O_3_ samples with the amount of 0.3 mL [C_4_Mim]BF_4_ also have stable photoactivity. After 3 months, the degradation rate of RHB has little change in k value (Figure 7D). An SEM image of the sample after placement for 3 months is shown in Figure S2. It shows that the morphology and structure of the as-synthesized α-Fe_2_O_3_ have few changes, which is very important for maintaining the photocatalytic activity of samples.

**Figure 8:**
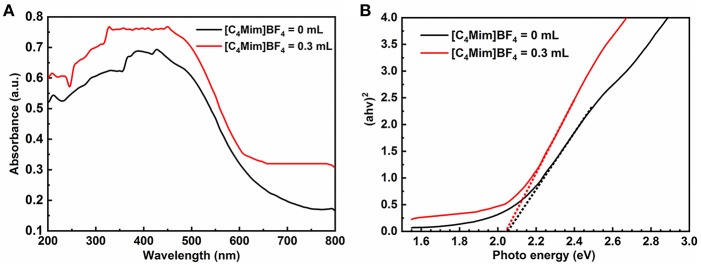
**(A)** UV-vis diffuse reflectance spectra and **(B)** plots of (αhv)^2^ vs. photon energy hv of the samples with addition = 0, 0.3 mL.

The photocatalytic activities of the α-Fe_2_O_3_ are closely related to its energy band. Figure 8A shows UV-vis diffuse reflectance spectra of α-Fe_2_O_3_ with additions of [C_4_Mim]BF_4_ = 0 and 0.3 mL in the wavelengths of 200–800 nm. Although the α-Fe_2_O_3_ particles and spheres show similar optical properties, the microsphere with a [C_4_Mim]BF_4_ addition of 0.3 mL shows stronger absorption, from 600 to 800 nm, resulting in a larger surface area that can absorb more light (Wang et al., 2011). Therefore, the as-prepared α-Fe_2_O_3_ microspheres exhibit better photocatalytic performance. Figure 8B is the plot of (αhv)^2^ vs. the energy of the absorbed light for α-Fe_2_O_3_. From Figure 8B, the band gap is determined to be 2.05 eV for the α-Fe_2_O_3_, indicating that the oxygen vacancies do not change the band gaps of α-Fe_2_O_3_ microstructures significantly.

Furthermore, in the process of photocatalytic reaction, ·OH is considered to be another main reaction species leading to the oxidative decomposition of organic pollutants. However, it was revealed that ·OH could not be generated during irradiation for Fe_2_O_3_ (Xiang et al., 2011). Because the photo-generated holes and electrons on Fe_2_O_3_ could not react with OH^−^/H_2_O and O_2_ to form ·OH and O^2−^, respectively, no ·OH can be generated in Fe_2_O_3_ (Xiang et al., 2011). This indicates that reactive species other than ·OH are present. However, several research results suggest that photo-generated holes and electrons can directly give rise to photocatalytic oxidation. As shown in this study, the photocatalytic performances of these α-Fe_2_O_3_ samples strongly depend on the [C_4_Mim]BF_4_ concentrations in the synthesis process. Therefore, it is likely that the high photocatalytic activity results from the novel structure, large surface area and strong absorption of visible light. Generally, large specific surface area provides more unsaturated coordination, which helps to improve the efficiency of electron hole separation (Tang et al., 2004). Furthermore, the increase of unsaturated coordination sites may improve the surface electron transfer rate. The increase of the surface electron transfer rate leads to the reduction of the probability of recombination and, therefore, the photo-generated charge carriers could more easily transfer to the surface to degrade the adsorbed RhB.

### Magnetic Properties

**Figure 9:**
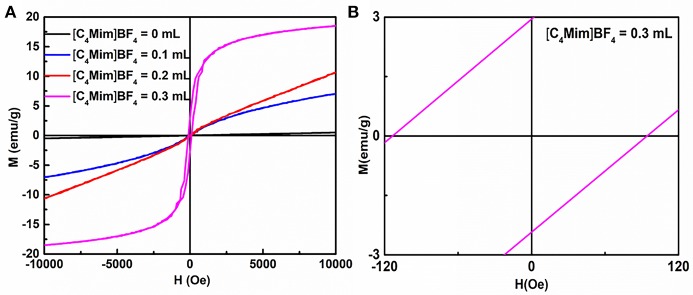
**(A)** Magnetization loops for α-Fe_2_O_3_ particles synthesized with different additions of [C_4_Mim]BF_4_. **(B)** A magnified view of curve.

It is well-known that α-Fe_2_O_3_ exhibits ferromagnetism (Sun et al., 2010). Figure 9 and Figure S3 shows the room temperature magnetic hysteresis loops and the magnetic field sweeping from −10.0 to 10.0 k Oe. Table 3 collects the values of remnant magnetization (Mr) and coercivity (Hc) of the as-synthesized α-Fe_2_O_3_ samples. It can be determined from the shape of the hysteresis loop that the synthesized α-Fe_2_O_3_ sample shows ferromagnetism. Additionally, the hysteresis loop did not reach saturation up to the maximum applied magnetic field, because of the presence of large-shape anisotropy (Bharathi et al., 2010). It is also shown in Table 3 that the residual magnetization and coercivity of assembled microspheres of α-Fe_2_O_3_ are large than α-Fe_2_O_3_ nanoparticles. It is widely understood that the morphology and structure of the as-synthesized samples will greatly affect the magnetization of ferromagnetic materials (Sorescu et al., 1999). Therefore, the assembly of the nano-sized and oriented particles and sheets into non-random structure results in the change of the single domain to the multidomain, leading to higher remnant magnetization and coercivity (Park et al., 2000). In summary, the difference can be attributed to the high crystallization, single domain size, surface (Tong et al., 2015), structure, and shape.

**Table 3:** The values of remnant magnetization (M_r_) and coercivity (H_c_) of the as-synthesized samples.

**Samples**	**0 mL**	**0.1 mL**	**0.2 mL**	**0.3 mL**
M_r_ (emu/g)	0.003	0.095	0.15	2.99
H_c_ (Oe)	32.72	41.99	51.97	100.44

## Conclusion

In summary, porous self-assembled α-Fe_2_O_3_ hollow microspheres were successfully prepared via a facile ionic liquid assistant synthesis approach. A nucleation–aggregation evacuation mechanism was the main formation of the porous hollow structures. EDA and [C_4_Mim]BF_4_ play significant roles on the formation of porous α-Fe_2_O_3_ hollow spheres. The increase in [C_4_Mim]BF_4_ promotes photocatalytic activities and α-Fe_2_O_3_ microspheres show higher photocatalytic activities. We believe that high specific surface area and porous hollow structure play an important role in improving the photocatalytic performance of as-prepared α-Fe_2_O_3_. Additionally, when the addition amount reaches 0.3 mL, the synthesized samples show ferromagnetism and can be easily recycled. The α-Fe_2_O_3_ hollow porous spheres prepared by our method have high photocatalytic activity, ideal ferromagnetic properties, and high specific surface area. They are expected to exhibit use as applications in sensors, catalysis, separation technology, environmental engineering, controlled drug delivery, and more.

## Data Availability

All datasets generated for this study are included in the manuscript and/or the Supplementary Files.

## Author Contributions

HY and YulZ partly designed the experiments and wrote the manuscript. QH, JZ, YuaZ, XX, and YL assisted in the analysis and interpretation of the data. JT and FW proposed the project and revised the manuscript.

### Conflict of Interest Statement

The authors declare that the research was conducted in the absence of any commercial or financial relationships that could be construed as a potential conflict of interest.
