# Supplementary material for: Controlled Synthesis of Hollow α-Fe2O3 Microspheres Assembled With Ionic Liquid for Enhanced Visible-Light Photocatalytic Activity
Source: Front Chem. 2019 Feb 27;7:58. doi: 10.3389/fchem.2019.00058 (PMC6402386; doi:10.3389/fchem.2019.00058)
Supplement: Supplementary file 1 [file Image_1.pdf]

Supplementary Material

Controlled Synthesis of Hollow α-Fe_2O_3 Microspheres Assembled with Ionic Liquid for Enhanced Visible-Light Photocatalytic Activity

Hang Yin[1 §], YuLing Zhao[1 §], Qingsong Hua[2], Jianmin Zhang[2], Yuansai Zhang[1], Xijin Xu[3], Yunze Long[1], Jie Tang[4]*, Fengyun Wang[1,5]*

*** Correspondence:** Jie Tang: TANG.Jie@nims.go.jp; Fengyun Wang: fywang777@163.com

Supplementary Figure

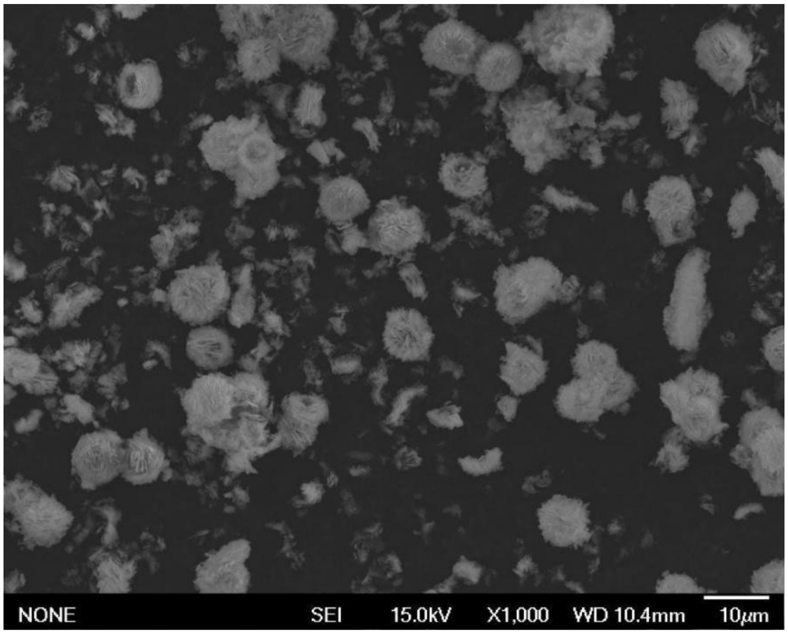

Figure S1 | SEM image of α-Fe_2O_3 sample (addition = 0.3 mL) placed after 3 months. The morphology and structure of the as synthesized sample is almost no change.

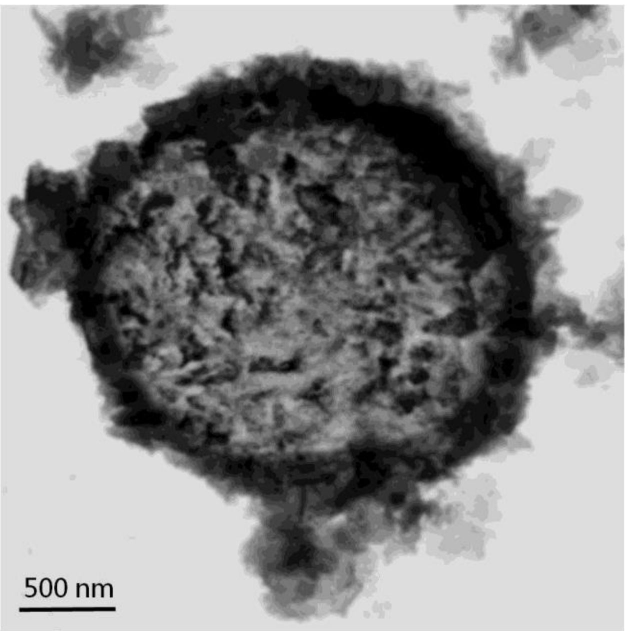

Figure S2 | TEM image of α-Fe_2O_3 hollow sphere with the addition of [C_4Mim]BF_4 = 0.2 mL.

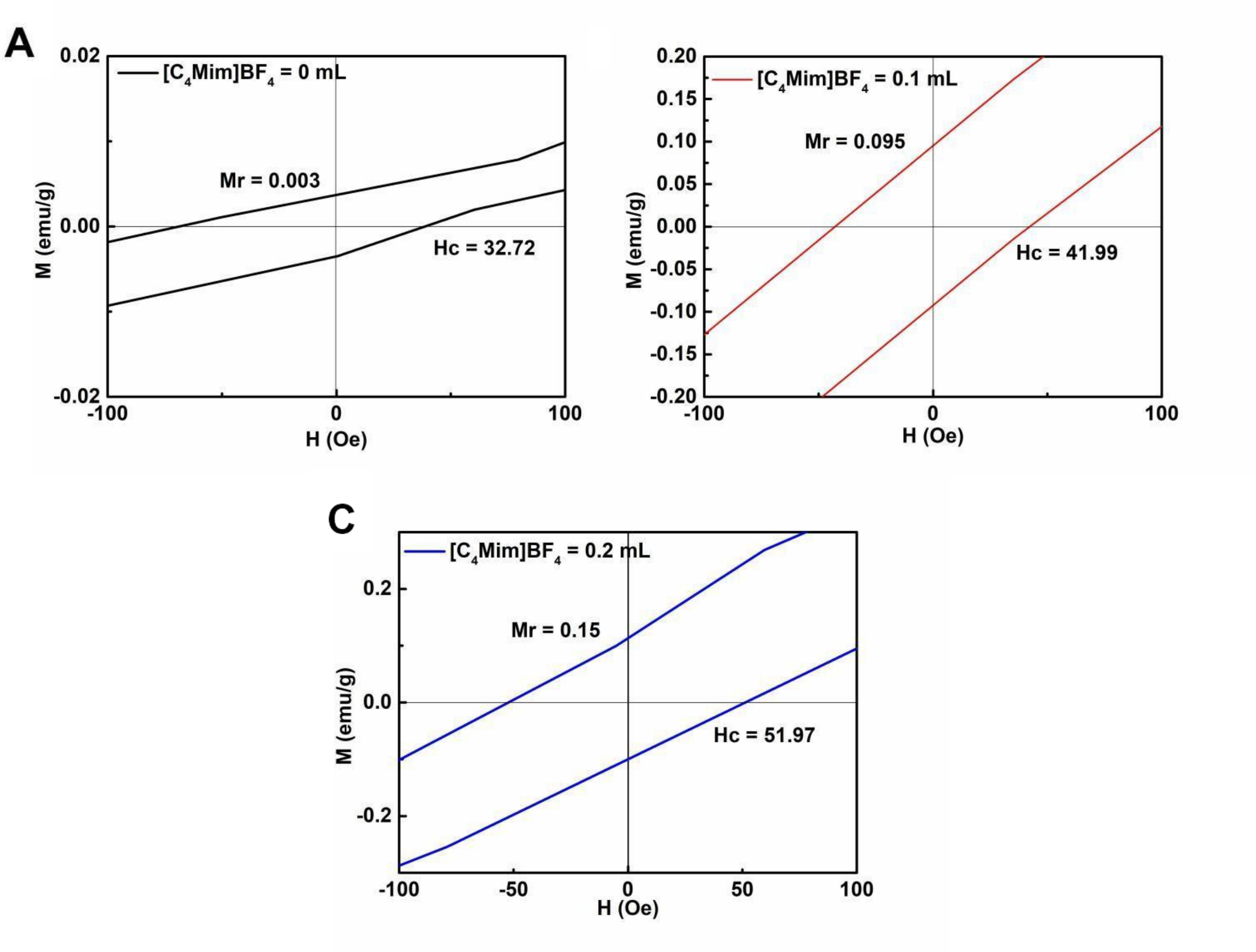

Figure S3 | Magnetization loops for α-Fe_2O_3 particles synthesized with different amounts of [C_4Mim]BF_4, (magnified view of curve). **(A)** [C_4Mim]BF_4 = 0 mL, **(B)** [C_4Mim]BF_4 = 0.1 mL, **(C)** [C_4Mim]BF_4 = 0.2 mL.
